# Effects of increased Kindlin-2 expression in bladder cancer stromal fibroblasts

**DOI:** 10.18632/oncotarget.17021

**Published:** 2017-04-10

**Authors:** Jitao Wu, Cuicui Yu, Li Cai, Youyi Lu, Lei Jiang, Chu Liu, Yongwei Li, Fan Feng, Zhenli Gao, Zhe Zhu, Shengqiang Yu, Hejia Yuan, Yuanshan Cui

**Affiliations:** ^1^ Department of Urology, The Affiliated Yantai Yuhuangding Hospital of Qingdao University, Yantai, Shandong, China; ^2^ Department of Anesthesiology, The Affiliated Yantai Yuhuangding Hospital of Qingdao University, Yantai, Shandong, China; ^3^ Department of Pathology, The Affiliated Yantai Yuhuangding Hospital of Qingdao University, Yantai, Shandong, China; ^4^ Department of Stem Cell Biology and Regenerative Medicine, Lerner Research Institute, Cleveland Clinic, Cleveland, OH, USA

**Keywords:** bladder cancer, Kindlin-2, cancer-associated fibroblasts, prognosis, invasion

## Abstract

Kindlin-2 is a focal adhesion protein highly expressed in bladder cancer stromal fibroblasts. We investigated the prognostic significance of Kindlin-2 in bladder cancer stromal fibroblasts and evaluated the effects of Kindlin-2 on the malignant behaviors of tumor cells. Immunohistochemical staining of 203 paraffin-embedded bladder cancer tissues showed that Kindlin-2 expression correlated with advanced stage, high grade, and relapse of bladder cancer. Kaplan-Meier survival analysis demonstrated that patients exhibiting high Kindlin-2 expression had shorter survival times than those with low Kindlin-2 expression (*p* < 0.01). Multivariate analysis revealed that high Kindlin-2 expression leads to poor prognosis in bladder cancer. Using cancer-associated fibroblasts (CAFs) isolated from human bladder cancer tissue, we observed that Kindlin-2 knockdown decreased CAFs activation, resulting in decreased expression of α-smooth muscle actin (α-SMA) and the extracellular matrix protein fibronectin. Kindlin-2 suppression also reduced CAF-induced bladder cancer cell migration and invasion. Moreover, we found that Kindlin-2 activates CAFs and promotes the invasiveness of bladder cancer cells by stimulating TGF-β-induced epithelial-mesenchymal transition. These results support targeting Kindlin-2 and the corresponding activated CAFs in bladder cancer therapy.

## INTRODUCTION

Bladder cancer is the second most common cancer of the genitourinary tract [1], and therapy fails in 33–75% of patients due to tumor recurrence and distant metastases [2]. Tumor stromal cells promote cancer cell migration and metastasis [3, 4]. Tumor tissue is not only composed of cancer cells but also various types of stromal cells, such as fibroblasts, macrophages, and endothelial cells [3, 5, 6]. Cancer-associated fibroblasts (CAFs) are activated fibroblasts, which are characterized by the expression of α-smooth muscle actin (α-SMA), fibroblast activation protein (FAP), fibronectin (FN), and vimentin [5, 7]. CAFs in the stroma microenvironment support growth and invasion of epithelia cells through secretion of cytokines, chemokines, and extra-cellular matrix (ECM) components [3, 4, 8]. Cytokines produced by CAFs promote the epithelial–mesenchymal transition (EMT), which is a major risk factor for metastasis [9, 10]. Specifically, Schulte et al. reported a correlation between CAFs and EMT markers in bladder cancer by immunohistochemistry (IHC) [11]. EMT is a process that allows an epithelial cell to acquire a mesenchymal phenotype through multiple biochemical changes, resulting in an increased migratory capacity [12].

Kindlin-2, also known as mig-2 (mitogen inducible gene-2), is one of three members in the Kindlin family of focal adhesion proteins [13]. Kindlin-2 promotes integrin activation, cell-ECM adhesion, myocardial formation [14], tissue homeostasis [15], and central nervous system development in embryos [16]. However, high stromal expression of Kindlin-2 is correlated with poor prognosis in patients with pancreatic ductal adenocarcinomas [17], and stimulates cancer progression and metastasis [18–20]. Increased Kindlin-2 expression in stromal cells is also positively correlated with higher grades of bladder cancer [21], indicating the protein may stimulate bladder cancer initiation and progression.

In our study, we analyzed Kindlin-2 protein expression in stromal fibroblasts from bladder cancer specimens, and investigated the effects of Kindlin-2 on CAF activation.

## RESULTS

### Clinicopathological factors associated with Kindlin-2 expression in bladder cancer tissues

**Figure 1:**
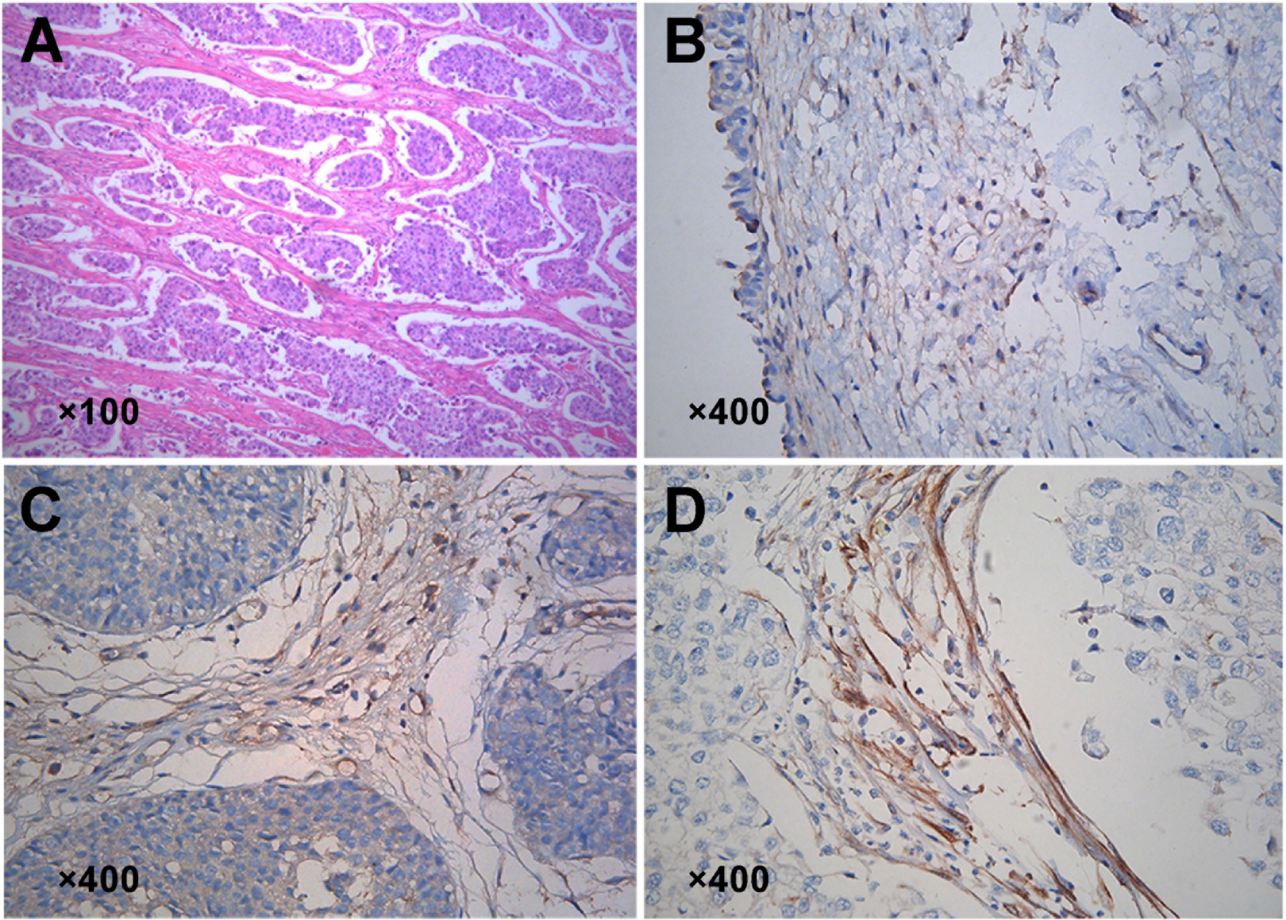
Expression of Kindlin-2 in bladder cancer and distant normal tissues (**A**) H&E staining showed stromal fibroblasts surrounding the cancer nets. (**B–D**) Detection of Kindlin-2 expression in stromal fibroblasts by IHC. (B) Normal bladder tissue. (C) Low grade bladder cancer tissue. (D) High grade bladder cancer tissue.

We first evaluated Kindlin-2 expression in 203 bladder urothelial carcinomas by immunohistochemistry (IHC). Stronger immunoreactivity was observed in the bladder cancer stromal fibroblasts compared to normal bladder tissue (Figure 1A–1D). Moreover, the staining of Kindlin-2 was moderate in the stromal fibroblasts of low-grade tumor tissues (Figure 1C), but strong in high-grade tissues (Figure 1D). Bladder cancer stromal fibroblast amount was classified as either low or high according to the median percentage of α-SMA positive areas (5%, interquartile range [IQR], 2–9%) for individual tumors. We then analyzed the correlation between clinicopathological parameters and Kindlin-2 expression in the 203 tumor tissue samples (Table 1). High expression of Kindlin-2 was detected in 109 of 203 cases (53.7%). Kindlin-2 expression did not show a significant correlation with age (*p* = 0.40), sex (*p* = 1.0), tumor number (*p* = 0.48), tumor size (*p* = 0.13), or stromal fibroblasts number (*p* = 0.26). However, high expression of Kindlin-2 in bladder cancer samples was positively correlated with high grade (*p* < 0.001), advanced stage (*p* = 0.011), and recurrence (*p* = 0.023).

**Table 1:** Correlation between Kindlin-2 expression and clinicopathological parameters in patients

Parameter	Case No.	Kindlin-2 expression		*p* value
		Low (%)	High (%)	
Age (years)				0.40
≤65	109	47 (43.1)	62 (56.9)	
>65	94	47 (50.0)	47 (50.0)	
Gender				1.0
Male	165	76 (46.1)	89 (53.9)	
Female	38	18 (47.4)	20 (52.6)	
Multifocality				0.48
Unifocal	118	52 (44.1)	66 (55.9)	
Multifocal	85	42 (49.4)	43 (50.6)	
Tumor size (cm)				0.13
≤ 3	140	70 (50.0)	70 (50.0)	
> 3	63	24 (38.1)	39 (61.9)	
Stage				0.011
Ta-1	88	50 (56.8)	38 (43.2)	
T2–4	115	44 (38.3)	71 (61.7)	
Grade				<0.001
Low	96	81 (84.4)	15 (15.6)	
High	107	13 (12.1)	94 (87.9)	
Recurrence				0.023
Yes	64	22 (34.4)	42 (65.6)	
No	139	72 (51.8)	67 (48.2)	
Stromal fibroblasts number				0.26
Low	101	51 (50.5)	50 (49.5)	
High	102	43 (42.2)	59 (57.8)	

### High Kindlin-2 expression in stromal fibroblasts predicts poor prognosis in bladder patients

**Figure 2:**
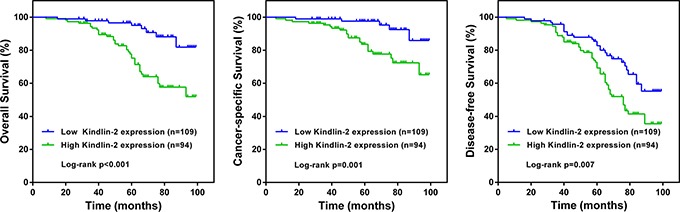
Kaplan-Meier survival curves according to Kindlin-2 expression in patients with bladder cancer **A.** Overall survival. **B.** Cancer-specific survival. **C.** Disease-free survival.

The median follow-up duration for these patients was 64 months (range 49 to 78). Kaplan-Meier survival curves and log-rank tests were performed to investigate the association between Kindlin-2 expression and the survival of bladder cancer patients. As presented in Figure 2A, patients with high Kindlin-2 expression had a worse overall survival (OS) than those with low Kindlin-2 expression (*p* < 0.001). Moreover, cancer-specific survival (Figure 2B) and disease-free survival (Figure 2C) were shorter in patients with high Kindlin-2 expression than those with low Kindlin-2 expression (both *p* < 0.01).

To identify whether Kindlin-2 expression was an independent prognostic factor in patients with bladder cancer, univariate and multivariate analyses were applied to OS. Univariate Cox regression analyses showed that Kindlin-2 expression (hazard ratio [HR] = 2.86, 95% confidence interval [CI] 1.57–5.23; *p* < 0.001), together with clinical TNM stage, histological grade, tumor recurrence, correlated to OS in bladder cancer (Table 2). Kindlin-2 expression (hazard ratio [HR] =1.73; 95% confidence interval [CI] 1.22–2.43; *p* = 0.026) was also an independent prognostic factor on multivariate analysis with the Cox regression model (Table 2).

**Table 2:** Univariate and multivariate analyses for overall survival in patients with bladder cancer

Variables	Univariate analysis			Multivariate analysis		
	Hazard ratio	95 % CI	*p* value	Hazard ratio	95 % CI	*p* value
Age (> 65y vs. ≤ 65y)	1.27	0.69–2.35	0.44			
Gender (male vs. female)	0.52	0.19–1.47	0.22			
Multifocality (multifocal vs. unifocal)	1.28	0.69–2.38	0.42			
Tumor size (> 3 cm vs. ≤ 3 cm)	0.73	0.39–1.36	0.32			
Stage (T2–4 vs. Ta-1)	4.49	2.08–9.71	< 0.001	5.21	1.78–15.31	< 0.001
Grade (high vs. low)	3.74	1.87–7.87	< 0.001	2.29	1.14–4.16	0.008
Recurrence (yes vs.no)	2.05	1.53–3.13	0.007	1.64	0.87–3.11	0.13
Kindlin-2 expression (high vs. low)	2.86	1.57–5.23	< 0.001	1.73	1.22–2.43	0.026
Stromal fibroblasts number (high vs. low)	1.22	0.93–1.59	0.18			

Abbreviations: CI: confidence interval.

### Kindlin-2 activates CAFs

**Figure 3:**
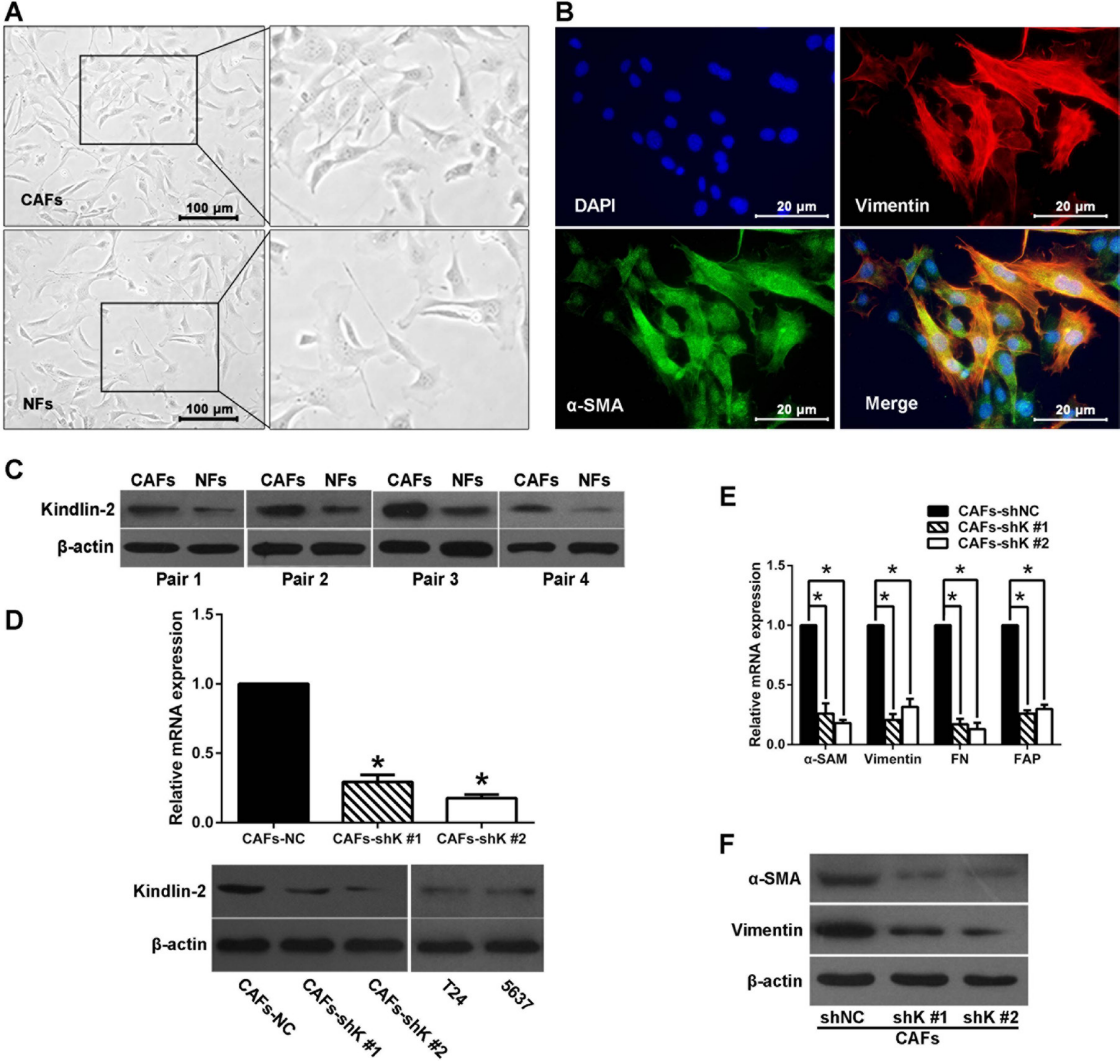
Kindlin-2 regulates fibroblast activation (**A**) Cancer-associated fibroblasts (CAFs) and normal fibroblasts (NFs) were freshly isolated from human bladder cancer tissue and maintained in FBS medium. (**B**) Double immunofluorescence staining of α-SMA (green) and vimentin (red) in CAFs. DAPI staining indicates cell nuclei. (**C**) Western blot shows Kindlin-2 protein level in four pairs of CAFs and corresponding NFs. (**D**) CAFs were transfected with Kindlin-2 shRNAs (shK #1 or #2) and non-silencing control shRNA (shNC). Kindlin-2 knockdown efficiency detected by qRT-PCR and western blot. Kindlin-2 expression in T24 and 5637 cells were also showed. (**E**) Vimentin, α-SMA, fibronectin, and fibroblast activation protein (FAP) mRNA expression is reduced in shK-treated CAFs, analyzed by qRT-PCR. (**F**) Western blot showing α-SMA and vimentin expression. (*p < 0.05).

Next we investigated whether Kindlin-2 regulates CAF activation. We isolated CAFs and normal fibroblasts (NFs) from human bladder cancer tissue, and maintained them in FBS medium (Figure 3A). The morphology of CAFs were elongated or stellated. We co-stained the vimentin (fibroblast marker) and α-SMA (smooth muscle marker) using immunofluorescence, and found most of the CAFs are double positive for vimentin and α-SMA (Figure 3B). This indicates that the CAFs are myofibroblasts, which is consistent with previous reports [4, 5, 8–10]. NFs isolated from normal bladder tissues showed decreased expression of vimentin and α-SMA compared with CAFs. Four pairs of primary CAFs and NFs were then used to examine Kindlin-2 expression by western blot. As shown in Figure 3C, increased Kindlin-2 expression was observed in CAFs compared with paired NFs.

CAFs were transfected with shRNAs targeting Kindlin-2 (shK) or a scrambled shRNA (shNC). Two different shRNAs targeting Kindlin-2 were used to exclude off-target effects. Both qRT-PCR and western blot (Figure 3D) showed that Kindlin-2 was knocked down in CAFs by both shRNAs (shK, #1 and #2). Compared with control, Kindlin-2 knockdown in CAFs resulted in decreased α-SMA, fibroblast activation protein (FAP), vimentin, and fibronectin (Figure 3E and 3F). These data suggest Kindlin-2 promotes the activation of CAFs in the bladder cancer microenvironment.

### Targeting Kindlin-2 in CAFs suppresses bladder cancer cell migration and invasion

**Figure 4:**
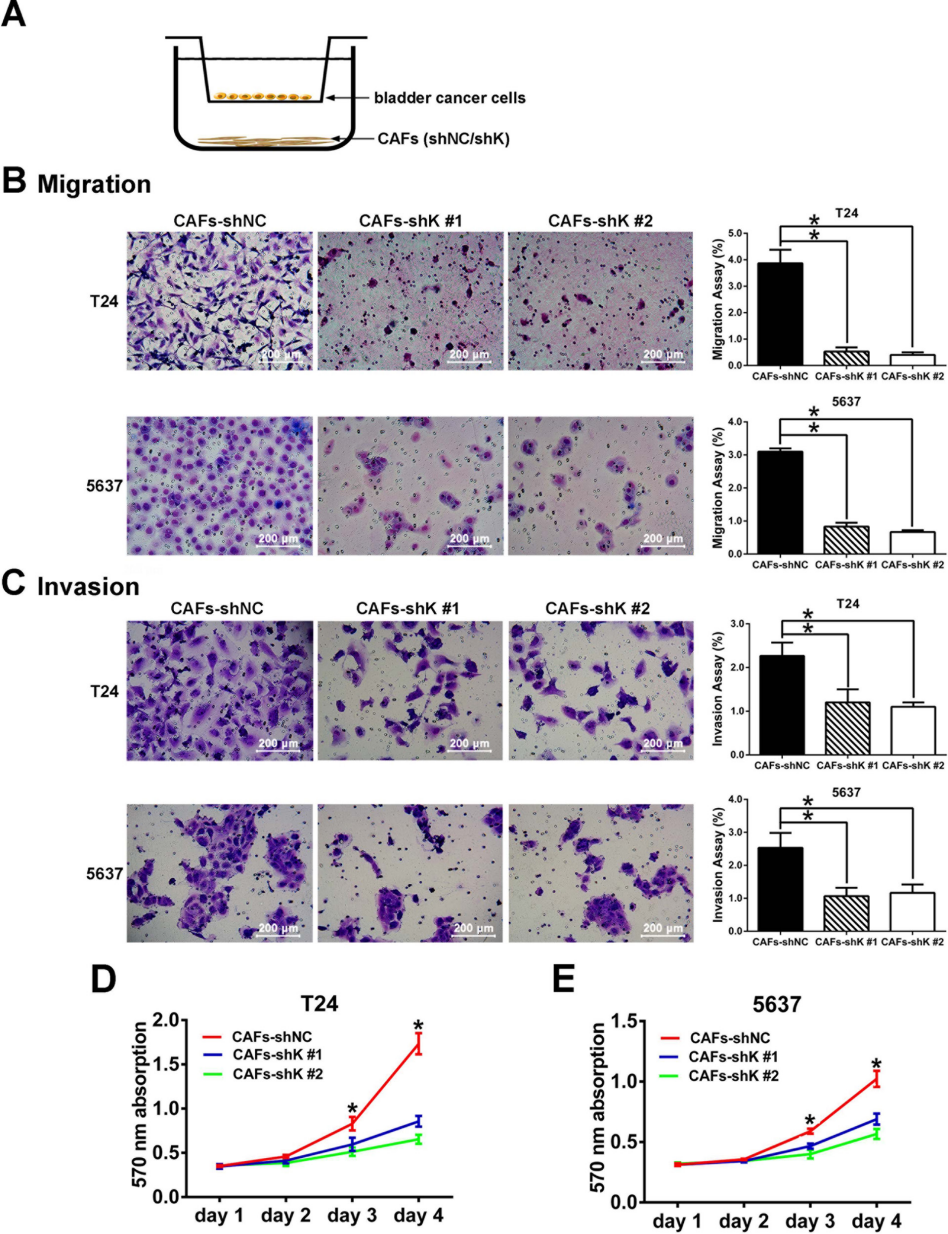
Kindlin-2 knockdown in CAFs inhibits bladder cancer cell migration and invasion (**A**) The transwell co-culture model: shRNA-treated cancer-associated fibroblasts (CAFs) were seeded into the lower chamber, and bladder cancer cells were put into the upper chamber. (**B**) Transwell migration assay was done by counting migrated T24 and 5637 cells co-cultured with shK-treated and shNC-treated CAFs. (**C**) Matrigel invasion assays of T24 and 5637 cells were analyzed in co-culture model with CAFs transfected with shK and shNC. The percentage of bladder cancer cells migrated was normalized to total number of cells on both sides of the chamber that were counted from identical but separate sets of chambers in the same condition. (**D** and **E**) MTT assay of T24 and 5637 cell growth. (*p < 0.05).

CAFs promote the growth and invasion of tumor cells through paracrine effects [3, 4, 8]. We examined whether CAFs can induce an attenuated effect on bladder cancer cell migration and invasion after Kindlin-2 knockdown. Using the stromal–epithelial co-culture strategy, T24 and 5637 bladder cancer cells were put into an upper chamber, and shRNA-treated CAFs were seeded into the lower chamber (Figure 4A). In the transwell experiments, both the T24 and 5637 cells co-cultured with shK-treated CAFs had fewer migrated and invaded cells than the cells co-cultured with shNC-treated CAF (Figure 4B and 4C). In addition, the MTT assay revealed that viability of bladder cancer cells decreased when cultured with shK-treated, CAF-conditioned media (Figure 4D and 4E). These data indicated that Kindlin-2 promotes CAF-induced bladder cancer cell migration and invasion.

### Kindlin-2 regulates bladder cancer cell migration and invasion via epithelial-mesenchymal transition signaling

**Figure 5:**
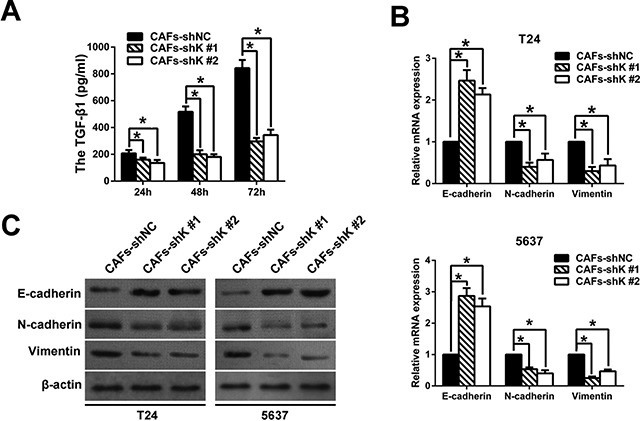
Kindlin-2 knockdown in CAFs changes the expression of EMT markers of co-cultured bladder cancer cells (**A**) TGF-β1 was determined in cancer-associated fibroblast (CAF) medium by ELISA. (**B**) Knocking down Kindlin-2 in CAFs increased E-cadherin expression, but inhibited N-cadherin and vimentin expression in co-cultured bladder cancer cells, as determined by qRT-PCR. (**C**) Western blot analysis of E-cadherin, N-cadherin, and vimentin expression in bladder cancer cells co-cultured with CAFs. β-actin was used as an internal control. (*p < 0.05).

CAFs secrete TGF-β, which enhances tumor invasion and metastasis [9, 22]. To investigate the mechanism of how Kindlin-2 influenced bladder cancer cell migration and invasion, we screened the TGF-β1 expression in CAFs by using an ELISA assay. CAF-secreted TGF-β1 was upregulated, and Kindlin-2 downregulation inhibited TGF-β1 expression (Figure 5A). These data suggest that TGF-β1 may stimulate CAF-induced cell migration and invasion.

TGF-β can accelerate the EMT of bladder cancer cells and induce cancer cell migration and invasion [9]. We examined the EMT marker changes in bladder cancer cells by co-culturing bladder cells with CAF-conditioned medium. After 72 h co-culture, we observed that Kindlin-2 knockdown decreased the mRNA expression of the epithelial marker E-cadherin, but increased expression of N-cadherin and vimentin (Figure 5B). Western blot confirmed that protein level changes of each marker mirrored the mRNA results (Figure 5C).

**Figure 6:**
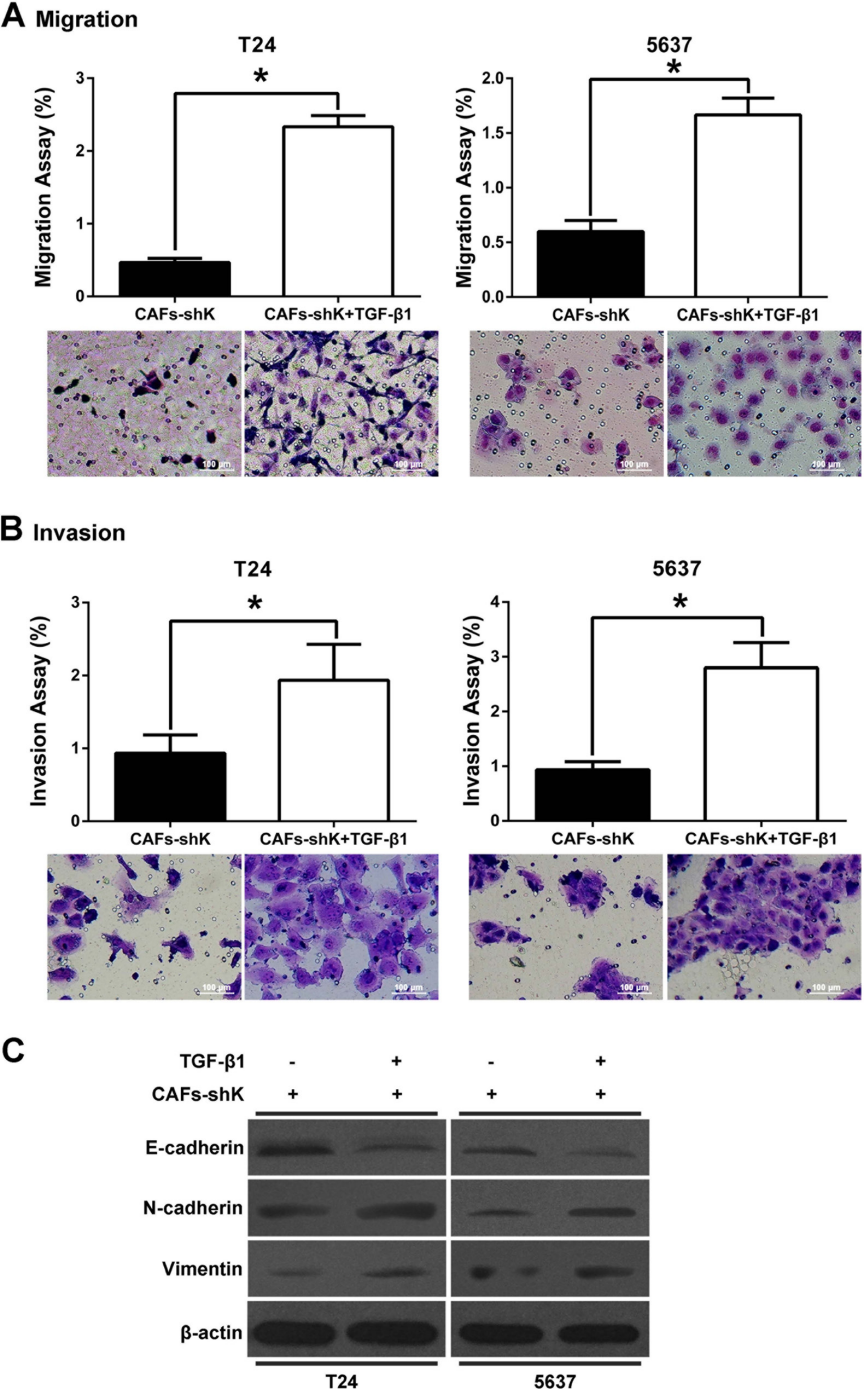
Kindlin-2 in CAFs regulates bladder cancer cell migration and invasion through mediating TGF-β Bladder cancer cells and shK-treated CFAs were co-cultured, and then treated with or without TGF-β1 (5 ng/ml). (**A** and **B**) Transwell assays were performed to test bladder cancer cell migration and invasion abilities. (**C**) Changes in protein expression levels of E-cadherin, N-cadherin, and vimentin were evaluated in co-cultured bladder cancer cells when treated with TGF-β1. β-actin was used as internal control. (*p < 0.05).

Decreased bladder cancer cell migration and invasion from Kindlin-2 knockdown can be rescued by addition of TGF-β1 (Figure 6A and 6B). Bladder cancer cells were co-cultured with shK-treated CAFs, then treated with TGF-β1 (5 ng/ml for 48 h). After TGF-β1 treatment, N-cadherin and vimentin expression increased, but E-cadherin expression decreased (Figure 6C and 6D).

## DISCUSSION

Kindlin-2 is a focal adhesion protein found in a variety of tissues [13–16], and can promote cancer invasion and metastasis [18–20, 23, 24]. In the present study, we found that Kindlin-2 protein expression in stromal cancer fibroblasts was correlated with advanced stages, high grades, and relapses of bladder cancer (Table 1). Kaplan-Meier survival curves and log-rank tests revealed that high Kindlin-2 expression was also correlated with the cancer-specific survival, disease-free survival, and overall survival of patients with bladder cancer (Figure 2). High Kindlin-2 expression is an independent prognostic factor for overall survival in patients with bladder cancer (Table 2). These correlations between Kindlin-2 expression and several clinicopathological parameters indicates that Kindlin-2 levels can be used as a marker to identify subsets of bladder cancer patients with more aggressive disease.

The lentivirus-mediated shRNAs targeting assay was used to knockdown Kindlin-2 expression in CAFs. Markers of CAF such as α-SMA, FAP, and vimentin were reduced upon knockdown of Kindlin-2 in CAFs. The expression of extracellular matrix protein fibronectin was also suppressed following depletion of Kindlin-2. These results suggest that Kindlin-2 may stimulate CAF activation in bladder cancer. Because Kindlin-2 appears to promote bladder cancer progression and invasion, it could also serve as a target for future cancer therapies.

We applied the co-culture method of cancer cells and CAFs, while CAFs were infected with Kindlin-2 shRNA and non-silencing control shRNA. The data showed that CAFs enhance the migration of T24 and 5637 cells, and Kindlin-2 knockdown in CAFs abolished CAF-induced cancer cell migration. This study demonstrated Kindlin-2 expression in CAFs is followed by bladder cancer cell migration and invasion, which is more evidence that targeting Kindlin-2 may provide a potential anti-metastasis strategy for bladder cancer.

Chemokines and cytokines secreted by CAFs can promote cancer cell growth and invasion [3, 4, 8, 25]. Zhuang et al. reported that CAFs expressed transforming growth factor-β1 (TGF-β1) at a higher level than normal fibroblasts, and TGF-β1 secreted by CAFs is an important factor to induce bladder cancer cell invasion [9]. In our study, downregulation of Kindlin-2 in CAFs was followed by decreased TGF-β1 production and subsequent secretion into the media. TGF-β signaling can induce bladder cancer cell motility and invasion, and associate with more frequent recurrence and decreased disease-specific survival of patients with bladder cancer [26]. In our study, TGF-β1secretion by CAFs decreased following Kindlin-2 knockdown, and cell migration and invasion capacity were suppressed. This study also showed that Kindlin-2 expression was upregulated by paracrine TGF-β1. These findings suggest that Kindlin-2 activates TGF-β signaling between CAFs and cancer cells, which promotes bladder cancer cell migration and invasion in the tumor microenvironment.

EMT is a process during which polarized epithelial cells acquire a migratory fibroblastoid phenotype, and it stimulates cancer invasion and metastasis [12]. Our previous study found that TGF-β induced downregulation of epithelial markers E-cadherin, β-catenin, and increasing expression of mesenchymal marker N-cadherin, which may be associated with the migration capability of bladder cancer cells [27]. In this study, the results showed that knocking down Kindlin-2 in CAFs led to increased E-cadherin expression, and decreased N-cadherin and vimentin expression in co-cultured bladder cancer cells. However, the inhibition effect in co-cultured bladder cancer cells following Kindlin-2 knockdown in CAFs can be rescued by addition of TGF-β1. Therefore, these data suggest Kindlin-2 signaling induces the EMT pattern in bladder cancer cells, and increases bladder cancer cell migration and invasion capacity. Kindlin-2 might contribute to the invasion of bladder cancer cells through upregulation of TGF-β expression, when CAFs interact with bladder cancer cells.

In summary, we demonstrated that high expression of Kindlin-2 in the cancer stroma defined a poor prognosis in patients with bladder cancer. Our data suggest that Kindlin-2 activates CAFs, which stimulates cancer cell migration and invasion by promoting the TGF-β-induced EMT process. These findings may highlight evidence to define Kindlin-2 and corresponding activated CAFs as potential therapeutic targets for bladder cancer.

## MATERIALS AND METHODS

### Patients and specimen tissue

We obtained bladder urothelial carcinoma tissues from patients who underwent transurethral resection of bladder tumor (TURB-t), radical cystectomy, or partial cystectomy for bladder cancer at our institution. We also obtained distant normal bladder tissue samples from paired carcinoma tissues as control. For immunohistochemical assays, we collected 203 paraffin-embedded bladder urothelial carcinoma samples from our hospital that were archived between May 1998 and May 2010. The criteria for enrollment were histopathological identification of bladder urothelial carcinoma, newly diagnosed without preoperative chemotherapy or radiotherapy, no history of other tumors, and good compliance for follow-up. Cancer stage was confirmed according to the 2002 American Joint Committee on Cancer (AJCC) Tumor-Node-Metastasis (TNM) staging system. The 2004 World Health Organization (WHO) classification criteria were used to categorize the histological grade of each tumor. This study was approved by the Ethics Committee of the Affiliated Yantai Yuhuangding Hospital of Qingdao University. Written informed consent was obtained from all patients who were enrolled in this study.

### Immunohistochemical staining and evaluation criteria

For immunohistochemistry (IHC), tumor sections were deparaffinized and rehydrated by routine methods, then incubated in 3% H_2_O_2_ for 30 min. Slides were incubated overnight at 4°C in a humidified chamber with diluted (1:500) mouse monoclonal antibody against Kindlin-2 (Santa Cruz, CA, USA), or with phosphate buffered saline (PBS) for control. All slides were then washed PBS three times, followed by incubation with secondary antibody. Sections were stained with 3, 3’-diaminobenzidine, and then counterstained with hematoxylin according to manufacturer protocols. The Kindlin-2 stained tissues were examined by two senior uropathologists based on the proportion and staining intensity of positively-stained peritumoral stroma cells. A score of 1 was given when ≤ 5% of cells were positive, 2 when 6% - 25%, 3 when 26%–50%, and 4 when ≥ 50% of cells were positive. Staining intensity was scored as 0 (negative), 1 (weak), 2 (moderate), and 3 (strong). The product of the two scores dichotomized Kindlin-2 expression as low (≤ 6) and high (> 6).

### Cell culture

Human bladder cancer cell lines T24 and 5637 were obtained from the Institute of Biochemistry and Cell Biology, Shanghai, China. Cells were grown in RPMI1640 (Gibco BRL, Grand Island, NY, USA) supplemented with 10% fetal bovine serum (FBS) (Gibco BRL, Grand Island, NY, USA) at 37 °C in a humidified incubator with 5% CO_2_. Fresh bladder cancer samples were obtained from the Affiliated Yantai Yuhuangding Hospital of Qingdao University. The sample collection and primary cell culture were approved by the Yantai Yuhuangding Hospital Ethics Committee.

CAFs and NFs were obtained as previously reported [4]. CAFs were defined as fibroblasts present within tumor masses, while NFs were defined by their association with normal epithelium more than 2 cm outside tumor masses. The specimen was cut into 2 to 3 mm^3^ pieces and suspended in RPMI-1640 medium containing 1mg/ml collagenase I (Sigma, St. Louis, MO, USA) and 1mg/ml hyaluronidase (Sigma, St. Louis, MO, USA) at 37°C for 5 h. The suspension was filtered and separated by the percoll gradient technique. The upper layer of cells was washed and cultured in RPMI-1640 medium containing 20% FBS. The CAFs are maintained in RPMI-1640 medium with 10% FBS. All CAFs used in the experiments had less than 10 passages.

### Immunofluorescence staining

The immunofluorescence staining was performed as previously described [4]. Cells were seeded into 4-well chamber slides and incubated for 24 h. The chamber slides with attached cells were fixed and blocked routinely, then incubated with vimentin and α-SMA antibodies (1:200; Sigma, St. Louis, MO, USA), then incubated with fluorescence-labeled secondary antibodies, and mounted by medium containing 4′, 6-diamidino-2-phenylindole (DAPI).

### RNA interference

To knockdown Kindlin-2 in CAFs, we employed the lentivirus infection strategy. A non-silencing control and a recombinant lentivirus expressing short-hairpin RNA (shRNA) targeting Kindlin-2 (target sequence: shRNA #1, 5′-GCCTCAAGCTCTTCTTGAT-3′; shRNA #2, 5′-GGAC AGTTCTTACAACTTA-3′) were constructed by GeneChem, Shanghai, China. Cells were also transfected with an empty vector control (data not shown). Cells were harvested for mRNA and protein analysis 3 days after infection.

### Quantitative real-time reverse transcription PCR (qRT-PCR)

Total RNA was extracted with Trizol reagent (Takara, Carlsbad, CA, USA) according to the manufacturer's protocol. The cDNA was synthesized using the RevertAid First-Strand cDNA Synthesis Kit (TransGene, Beijing, China). For qRT-PCR, each sample was analyzed in triplicate with amplification conditions of 1 cycle at 95°C for 10 min, then 45 cycles of 95°C for 15 s and 60°C for 60 s. The relative expression of mRNA was calculated by using the formula 2^-∆∆CT^. Primer sequences were as follows: Kindlin-2: sense, 5′-AACCAAGGATGGCTTGATTCC-3′; antisense, 5′-CAGGGCTGCAAACATCATCAT-3′; α-SMA: sense, 5′-GGCAAGTGATCACCATCGGA-3′; antisense, 5′-GTGGTTTCATGGATGCCAGC-3′; vimentin: sense, 5′-TGGACCAGCTAACCAACGAC-3′; antisense, 5′-GCC AGAGACGCATTGTCAAC-3′; fibronectin (FN): sense, 5′-CAGTAGACCTGTGGAGCATTAC-3′; antisense, 5′-CGGCTCCACATCCTCAATAA-3′; and fibroblast activation protein (FAP) sense, 5′-GGCTTATCACCTGA TCGGCAA-3′; antisense, 5′-TTTACTCCCAACAGGC GACC-3′; β-actin: sense, 5′-GGCGGCACCACCATGTA CCCT-3′; antisense, 5′-AGGGGCCGGACTCGTCATACT-3′.

### Cell migration and invasion assay

Cell invasion and migration assays were performed in 24-well transwell polycarbonate membrane inserts with 8-μm pores (Corning Life Sciences, Bedford, MA, USA), with or without Matrigel (BD BioSciences, San Jose, CA, USA). Bladder cancer cells (5 × 10^4^) in serum-free RPMI 1640 medium were plated in the upper chamber with or without Matrigel in triplicate. CAFs (1 × 10^5^) transfected with shNC or shK were seeded into the lower chamber in RPMI 1640 medium supplemented with 5% FBS. The invaded and migrated cells were counted after the cells had been individually incubated for 24 and 10 hours, respectively. Nonpenetrating cells were removed from the upper surface of the filter with a cotton swab. For quantification, those that had migrated or invaded to the bottom surface were fixed, stained and scored visually in 5 random fields under a microscope. Each experiment was performed in replicates, and the mean value was calculated from three independent experiments.

### MTT assay

Bladder cancer cells were seeded into 96-well plates at 5000/well and incubated overnight. Conditioned cell media from CAFs was then transferred onto bladder cancer cells for 48 h. At the end of treatment, 10 μl (5 mg/ml) of 3-(4,5-Dimethylthiazol-2-yl)-2,5-diphenyltetrazolium bromide (MTT) (Sigma, St. Louis, USA) was added to each well. After 150 μl of dimethyl sulfoxide (DMSO) was added, the optical density (OD) of the DMSO solution was measured at 570 nm using a microplate reader (Bio-Rad, Hercules, CA, USA). The cell viability was calculated as follows: Cell survival rate (%) = Treatment group A570/Control group A570×100%. All assays were done with 6 parallel samples.

### ELISA assay

Quantification of TGF-β levels in the medium of co-cultured cells were calculated by ELISA assay according to the manufacturer's instructions (BOSTER, Wuhan, China). Plates were tapped dry, TGFβ1 antibody was added, and the plates incubated for 60 min at 37 °C. After being washed with PBS, tetramethylbenzidine (TMB) substrate was added to each well. After incubation in the dark for 30 min at 37°C, 100 μl TMB stop buffer was used to stop the reaction. Negative control samples were incubated only with secondary antibody. The plates were read at 450 nm via microplate reader.

### Western blot

Protein lysates were analyzed by 12% sodium dodecyl sulfate-polyacrylamide gel electrophoresis (SDS-PAGE), and were subsequently transferred to nitrocellulose membranes (Millipore, Darmstadt, Germany). After blocking with 5% nonfat milk in Tris-buffered saline with Tween for 1 h, the membrane was incubated with primary antibody at 4°C overnight. The primary antibodies used for western blot were anti-Kindlin-2 antibody (1:3000; Santa Cruz, CA, USA), α-SMA (1:2000; Sigma, MO, USA), vimentin (1:2000; Santa Cruz, CA, USA), E-cadherin (1:300; Abcam, Cambridge, UK), N-cadherin (1:1000; Cell Signalling, MA, USA) and β-actin (1:2000; Santa Cruz, CA, USA). After three ten-minute washes with Tris-buffered saline with Tween, the membrane was incubated with HRP-conjugated secondary antibodies (1:5000; Santa Cruz, CA, USA) for 1 h at room temperature. Immunoreactive bands were detected by a chemiluminescent detection system (ECL, Pierce, Rockford, IL, USA).

### Statistical analysis

Data are presented as mean ± SD of three independent experiments. Differences between groups were analyzed with Student's *t-test* for unpaired observations. Chi-square test was used to evaluate the results of IHC staining. Survival curves were plotted by the Kaplan-Meier method and compared using the log-rank test. The significance of covariates was analyzed by the Cox proportional hazards model in univariate and multivariate analysis. Statistical significance threshold was *p* < 0.05. All statistical analyses were performed by SPSS 19.0 (Chicago, IL, USA).
